# Total Testosterone Measured by Liquid Chromatograph‐Tandem Mass Spectrometry Refines Diagnosis of Biochemical Hyperandrogenism and Better Identifies Subgroup at Genuine Risk of Adverse Fertility Outcomes in Women With Polycystic Ovary Syndrome

**DOI:** 10.1002/rmb2.70013

**Published:** 2026-01-28

**Authors:** Jian Li, Qi Wu, Jing Cong, Hui Chang, Hong‐Li Ma, Duo‐Jia Zhang, Yu Wang, Rui‐Zhe Zhang, Richard K. T. Kam, Chung Shun Ho, Michael Ho Ming Chan, Ronald Ching Wan Ma, Ernest Hung Yu Ng, Ben Willem J. Mol, Elisabet Stener‐Victorin, Richard S. Legro, Chi Chiu Wang, Xiao‐Ke Wu, PCOSAct Study Group

**Affiliations:** ^1^ Department of Obstetrics and Gynecology, Affiliated Hospital Guizhou Medical University Guiyang China; ^2^ Department of Obstetrics and Gynecology, First Affiliated Hospital Heilongjiang University of Chinese Medicine Harbin China; ^3^ Department of Obstetrics and Gynaecology The Chinese University of Hong Kong Hong Kong China; ^4^ Department of Research and Development Shenzhen Coreion Biotechnology Co., Ltd Shenzhen China; ^5^ Center for Reproductive Medicine, Henan Key Laboratory of Reproduction and Genetics The First Affiliated Hospital of Zhengzhou University Zhengzhou China; ^6^ Department of Chemical Pathology Prince of Wales Hospital Hong Kong China; ^7^ Department of Medicine and Therapeutics The Chinese University of Hong Kong Hong Kong China; ^8^ Li Ka Shing Institute of Health Sciences and School of Biomedical Sciences The Chinese University of Hong Kong Hong Kong China; ^9^ Department of Obstetrics and Gynaecology The University of Hong Kong Hong Kong China; ^10^ Department of Obstetrics and Gynaecology, Monash Medical Centre Monash University Clayton Victoria Australia; ^11^ Department of Physiology and Pharmacology Karolinska Institutet Stockholm Sweden; ^12^ Department of Obstetrics and Gynecology Pennsylvania State University Hershey Pennsylvania USA; ^13^ Chinese University of Hong Kong‐Sichuan University Joint Laboratory in Reproductive Medicine The Chinese University of Hong Kong Hong Kong China; ^14^ The First Affiliated Hospital of Zhejiang Chinese Medical University Hangzhou China

**Keywords:** biochemical hyperandrogenism, liquid chromatograph‐tandem mass spectrometry, polycystic ovary syndrome, total testosterone

## Abstract

**Purpose:**

To compare total testosterone (TT) measured by liquid chromatograph‐tandem mass spectrometry (LC–MS/MS) with electro‐chemiluminescent immunoassay (ECLIA) in the diagnosis and management of infertile women with polycystic ovary syndrome (PCOS).

**Methods:**

Baseline TT was measured by LC–MS/MS and ECLIA in 906 infertile women with PCOS. The associations of TT from both methods with clinical phenotypes and fertility outcomes were estimated; relative risk (RR) and 95% confidence intervals (CIs) were computed. Subgroup analysis was conducted according to the TT levels.

**Results:**

The average TT levels measured by ECLIA were higher than those measured by LC–MS/MS (mean percentage difference 23.8%, 95% limits of agreement −44.2% to 91.9%). When biochemical hyperandrogenism (HA) defined as TT ≥ 1.7 nmol/L by LC–MS/MS method, a higher proportion of patients were identified having biochemical HA using ECLIA (44.0% vs. 24.0%, *p* < 0.001) than LC–MS/MS. Only those with TT levels ≥ 1.7 nmol/L measured by LC–MS/MS had an increased risk of adverse fertility outcomes compared to patients with normal TT levels, including ovulation, preterm labor, and neonatal intensive care unit.

**Conclusion:**

Our findings indicated that LC–MS/MS refined the diagnosis of biochemical hyperandrogenism and better identified the subgroup at genuine risk of adverse fertility outcomes in infertile women with PCOS.

**Trial Registration:**

The NIH Clinical Trial Registry number: NCT01573858 and Chinese Clinical Trial. Registry number: ChiCTR‐TRC‐12002081

## 1 Introduction

Polycystic ovary syndrome (PCOS) is one of the most common endocrine disorders affecting individuals of reproductive age, with a prevalence that varies between 11% and 13% depending on the diagnostic criteria and geographical regions [1, 2]. Androgen excess, a critical characteristic of PCOS, is evident in up to 85% of patients with PCOS [3]. However, its clinical manifestations including hirsutism, acne, and alopecia are less frequently observed. In addition to impairing ovulation [4], androgen excess also adversely impacts fertility and pregnancy outcomes in this population [5], and can even increase the risk of PCOS development in their female offspring [6]. Consequently, the biochemical assessment of circulating androgens to evaluate biochemical hyperandrogenism (HA) is essential and indispensable, yet making an accurate diagnosis of HA is still a big challenge.

Currently, the determination of biochemical HA is commonly based on circulating concentrations of total testosterone (TT) and free testosterone (FT), or calculation of the free androgen index (FAI) given sex hormone‐binding globulin (SHBG). Although TT has been extensively utilized, its accuracy is primarily influenced by the assay methods employed [7], which include direct radioimmunoassay (RIA), electro‐chemiluminescent immunoassay (ECLIA), and liquid chromatograph‐tandem mass spectrometry (LC–MS/MS). FT refers to another crucial laboratory indicator of HA [7], yet the equilibrium dialysis method used in its measurement would affect the assay's performance [8]. FAI, calculated as the ratio of TT to SHBG, is well correlated with HA and fertility outcomes and is preferable for usage in this population. However, a reduced SHBG level, which may result from certain disorders, such as inflammation, can lead to inaccurate FAI values.

According to the International Evidence‐Based Guideline for the assessment and management of PCOS 2023, LC–MS/MS is a conditional recommendation to measure TT in assessment of biochemical HA, particularly for detecting mild androgen excess [1]. In contrast to ECLIA, it has been reported that TT measured by LC–MS/MS had increased accuracy [9] and superior specificity [10], and positively correlates with clinical HA [11], while TT concentrations quantified are significantly lower by LC–MS/MS [12]. Despite the recommendation of TT measurement by LC–MS/MS for the diagnosis and management of PCOS [13], the quality of evidence regarding LC–MS/MS used to assess biochemical hyperandrogenism is still low and the normative cut‐off value is uncertain, due to small sample size and inconsistent inclusion criteria. What's more, this method does not significantly enhance diagnostic capabilities compared to alternative approaches [12]. Until now, the majority of studies about LC–MS/MS focus on diagnosis of biochemical HA, while a few inadequately investigate the associations between TT levels measured by LC–MS/MS and clinical features, and fertility and obstetric outcomes remain unexplored within the context of this condition.

This study aims to thoroughly compare the association of TT quantified by ECLIA and LC–MS/MS with clinical and fertility and obstetric outcomes in patients with PCOS who underwent ovulation induction, and further conduct a subgroup analysis using TT cut‐off value of 1.7 nmol/L measured by these two methods to identify a subgroup at genuine risk of adverse fertility outcomes.

## 2 Material and Methods

This prospective study was designed to assess the utility of baseline total testosterone levels, as measured by liquid chromatograph‐tandem mass spectrometry (LC–MS/MS), in participants with PCOS sourced from the Polycystic Ovary Syndrome Acupuncture plus Clomiphene Trial (PCOSAct) [14]. Ethical approval for the study was granted by the Ethics Committee at the First Affiliated Hospital of Heilongjiang University of Chinese Medicine (IRB 2010HZYLL‐010), and the trial was registered on chictr.org.cn (ChiCTR‐TRC‐12002081). PCOS diagnosis adhered to the modified Rotterdam criteria tailored for the Chinese population, which included oligomenorrhea (defined as menstrual interval > 35 days and < 8 cycles per year) or amenorrhea (defined as menstrual interval > 90 days) as mandatory criteria, in conjunction with either clinical or biochemical evidence of hyperandrogenism or the presence of polycystic ovaries (diagnosed by transvaginal ultrasound when at least one ovary had a volume of > 10 mL or there were 12 or more follicles measuring 2–9 mm in diameter). Within the trial of PCOSAct, 1000 eligible participants who had not undergone hormone treatment within the preceding 6 months were randomly allocated to receive either clomiphene or placebo, with or without acupuncture therapy. Clomiphene administration started at an initial dose of 50 mg, with the dosage incrementally increased up to a maximum of 150 mg. Active acupuncture involved deep needle insertion complemented by both manual and low‐frequency electrical stimulation, lasting 30 min and administered twice weekly. Blood samples were collected following an overnight fast on the first day post‐randomization subsequently centrifuged and frozen at the local sites, and then transported and stored in the core laboratory of Heilongjiang University of Chinese Medicine for a period of up to 3 years before assay. Assays were performed after the completion of participant enrollment. Written informed consent for the utilization of baseline blood samples for this study was procured from all participants. A total of 906 serum samples were available for analysis in this study.

Baseline demographic data encompassed age, weight, height, waist and hip circumference, blood pressure, skin and hair condition. The body mass index (BMI) was computed by the formula: BMI = weight (kg)/[height (m)]^2^. According to the criteria established for the Chinese population [15], overweight was defined as BMI of ≥ 24 kg/m^2^ and < 28 kg/m^2^, while obesity was classified as BMI ≥ 28 kg/m^2^. Hirsutism, namely clinical hyperandrogenism, was diagnosed when the modified Ferriman‐Gallwey (mFG) score was ≥ 5 [16]. Acne was identified as present with an acne score greater than zero, assessed using a standardized acne lesion assessment diagram and definition [17]. Acanthosis score (AS) was evaluated by the neck severity scale [18]. Metabolic syndrome (MetS) was diagnosed following the updated criteria of the China Diabetes Society (CDS) [19].

Sex hormones, including luteinizing hormone (LH), follicle‐stimulating hormone (FSH), TT (the sensitivity range, intra‐assay coefficient of variability (CV) and inter‐assay CV were 0.025–15.0 ng/mL, 4.7 and 8.4), FT, SHBG, estradiol (E2), and progesterone (P4), were quantified by ECLIA (Roche Diagnostics GmbH, Mannheim, Germany), expect FT, which was measured by radioimmunoassay (DSL‐5400, Diagnostic Systems Laboratories, Webster, TX). Anti‐Mullerian hormone (AMH) was assessed by an Ultra‐Sensitive AMH ELISA assay (Ansh Lab, Wester, TX, USA). Metabolic profiles, including fasting glucose and insulin, total cholesterol (TC), triglyceride (TG), high‐density lipoprotein (HDL) and low‐density lipoprotein (LDL), were also evaluated. Fasting glucose was measured by hexokinase assay (Maker, Chengdu, China), while insulin was quantified by ECLIA (Roche Diagnostic, Basel, Switzerland). TC and TG levels were determined by the N‐(3‐sulfopropyl)‐3‐methoxy‐5‐methylaniline method (Wako Diagnostics). HDL and LDL were measured by direct‐method assays. The free androgen index (FAI), calculated as TT to SHBG, was derived using the formula TT*100/SHBG in %. The homeostasis model assessment of insulin resistance (HOMA‐IR) was computed using the formula: [HOMA‐IR = (fasting insulin × fasting glucose)/22.5].

Baseline serum TT, as measured by LC–MS/MS, was quantified using a Waters I‐class Xeyo TQ‐S liquid chromatography‐mass spectrometry with an electrospray ionization source in positive‐ion mode (Waters Corporation, Milford, MA, USA). The internal standard utilized was stable isotope‐labeled testosterone (T‐3C13) prepared in a 35% v/v methanol solution. Calibration was performed using the 6PLUS1 Multilevel Serum Calibrator Set MassChrom Steroid Panel 2 (Gräfelfing, Munich, Germany). LC–MS/MS conditions were with electrospray ionization source, capillary voltage at 3.0 kV, source temperature of 150°C. The desolvation temperature was of 500°C. TT was extracted from 100 μL serum via liquid–liquid extraction. Briefly, serum sample was mixed with 50 𝑢L of internal standard mixture containing T‐3C13, and was extracted using 0.6 mL tert‐butyl‐methyl‐ether (MTBE) at room temperature. The organic supernatant was divided into 2 equal portions in two microcentrifuge tubes. Then the MTBE extract was vacuum dried and reconstituted in 150 𝑢L of 35% v/v methanol and subjected to LC–MS/MS analysis. The total imprecision of the assay was < 9% (< 3% when excluding the lower limit of quantitation, LLOQ), and the inaccuracy was < 10% across the entire concentration range (< 7% when excluding LLOQ). Overall, the LC–MS/MS assay employed was suitable for the quantitation of endogenous steroids at both physiological and pathophysiological levels in serum samples. The biochemical hyperandrogenism was defined as TT levels of ≥ 1.7 nmol/L quantified by LC–MS/MS, and this threshold was established using LC–MS/MS method based on 180 healthy pre‐menopausal Chinese individuals with features of PCOS [20].

Ovulation was determined by the presence of at least one serum progesterone level indicative of ovulation, measured weekly throughout a cycle, with a threshold of > 9.54 nmol/L (3 ng/mL). Ovulation rates per participant were calculated as the ratio of cumulative ovulatory participant to the total number of participants. An ovulatory participants was defined as one who experienced at least one ovulatory cycle during the four treatment cycles. Conception was defined as a positive serum human chorionic gonadotropin test, as determined by the local site. Clinical pregnancy was confirmed by the detection of an intrauterine pregnancy with fetal heart pulsation via transvaginal ultrasound. Live birth was defined as the delivery of a viable infant with a gestational age of ≥ 20 weeks. Obstetric outcomes including threatened miscarriage, gestational diabetes mellitus (GDM), preterm labor, and neonatal intensive care unit (NICU) admission rate, were also recorded.

Data were described as mean (standard deviation, SD) for continuous variables or as frequencies (percentages) for categorical variables. Mann–Whitney *U* test was used to compare differences between groups. Passing‐Bablok regression analysis was employed for pairwise comparisons of assay methodologies. Bland–Altman plots were performed to visually assess the linearity of the measurement method comparisons and to evaluate the homoscedasticity of measurement variability, with the mean difference (MD) and 95% limits of agreement (LOA) being computed. The correlation between TT and clinical manifestations, fertility, and obstetric outcomes was determined by Spearman's rank correlation. Relative risk (RR) and 95% confidence interval (CI) were computed by modified Poisson regression [21], adjusted for age, BMI, and intervention, due to that variables were demonstrated to have effects on fertility and obstetric outcomes. Subgroup analysis was conducted based on TT levels with a threshold of 1.7 nmol/L, with patients categorized into three groups: < 1.7 nmol/L by both methods, ≥ 1.7 nmol/L by ECLIA but < 1.7 nmol/L by LC–MS/MS, and ≥ 1.7 nmol/L by both methods. A two‐tailed *p* value of less than 0.05 was considered as statistical significance. Statistical analyses were performed in R Version 3.6.1 (http://www.rstudio.com/).

## 3 Results

In total, 906 infertile patients with PCOS were included in the study, with a mean age of 27.3 years, mean BMI of 25.7 kg/m^2^, mean mFG score of 3.0, mean acne score of 0.4 and mean acanthosis nigricans score of 1.3. The baseline demographic parameters, hormone and metabolic profiles, and treatment are detailed in Table 1.

**TABLE 1:** Baseline clinical characteristics in patients with PCOS undergoing ovulation induction.

Baseline characteristics	Mean (SD) or *n* (percent)
Anthropometric parameters	
Age (years)	27.3 ± 3.3
BMI (kg/m^2^)	25.7 ± 4.7
Waist circumference (cm)	88.6 ± 12.1
Hip circumference (cm)	100.8 ± 9.1
Systolic blood pressure (mmHg)	114.3 ± 8.6
Diastolic blood pressure (mmHg)	75.5 ± 7.9
Modified FG score	3.0 ± 2.9
Acne score	0.4 ± 0.7
Acanthosis score	1.3 ± 0.6
Hormones levels	
LH (IU/L)	11.4 ± 5.3
FSH (IU/L)	6.1 ± 1.5
E2 (pmol/L)	217.7 ± 95.1
P4 (nmol/L)	2.8 ± 6.8
FT (pmol/L)	8.5 ± 2.8
AMH (ng/mL)	13.5 ± 5.9
SHBG (nmol/L)	34.7 ± 26.5
TT (nmol/L)	
From ECLIA	1.8 ± 0.6
From LC–MS/MS	1.5 ± 0.7
TT to SHBG ratio (%)	
From ECLIA	7.6 ± 5.4
From LC–MS/MS	5.7 ± 3.5
Metabolic parameters	
Fasting insulin (pmol/L)	103.8 ± 76.1
Glucose (mmol/L)	5.0 ± 0.9
HOMA‐IR index	3.3 ± 2.6
Total cholesterol (mmol/L)	4.9 ± 1.1
Triglyceride (mmol/L)	1.7 ± 1.0
High‐density lipoprotein (mmol/L)	1.2 ± 0.3
Low‐density lipoprotein (mmol/L)	3.1 ± 0.9
Treatment, *n* (%)	
Active acupuncture plus clomiphene citrate	227 (25.1)
Control acupuncture plus clomiphene citrate	229 (25.3)
Active acupuncture plus placebo	226 (24.9)
Control acupuncture plus placebo	224 (24.7)

*Note:* The referent range of partial variables: TT from ECLIA 0.29–1.67 nmol/L; TT from LC–MS/MS < 1.7 nmol/L; free testosterone 0–3.09 pg/mL, SHBG 18–114 nmol/L, AMH 13.5–2240 pg/mL.

Abbreviations: AMH, anti‐Mullerian hormone; BMI, body mass index; E2, estradiol; FSH, follicle stimulating hormone; FT, free testosterone; LH, luteinizing hormone; P4, progesterone; SHBG, sex hormone binding globulin; TT, total testosterone.

**FIGURE 1:**
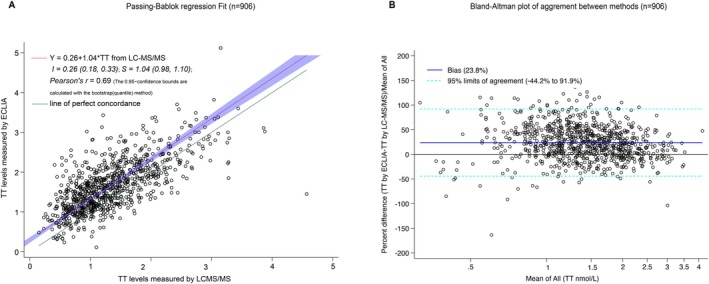
Passing‐Bablok regression and Bland–Altman graphs. (A) Passing‐Bablok regression graph with depiction of the 95% confidence interval (CI) for both the slope and intercept. Regression line equation (solid red lines): Y = 0.26 + 1.04 x. The 95% CI for intercept spans from 0.18 to −0.33, while for the slope, it ranges from 0.98 to 1.10. These intervals suggest a good agreement between the datasets. (B) The Bland–Altman plot illustrates the mean percentage difference (sold blue line) and the 95% limits of agreement (dashed light green lines). The plot indicates that the mean percentage difference (bias) was 23.8% (95% limits of agreement −44.2% to 91.9%) between ECLIA and LC–MS/MS. ECLIA, electro‐chemiluminescent immunoassay; LC–MS/MS, liquid chromatograph‐tandem mass spectrometry.

**FIGURE 2:**
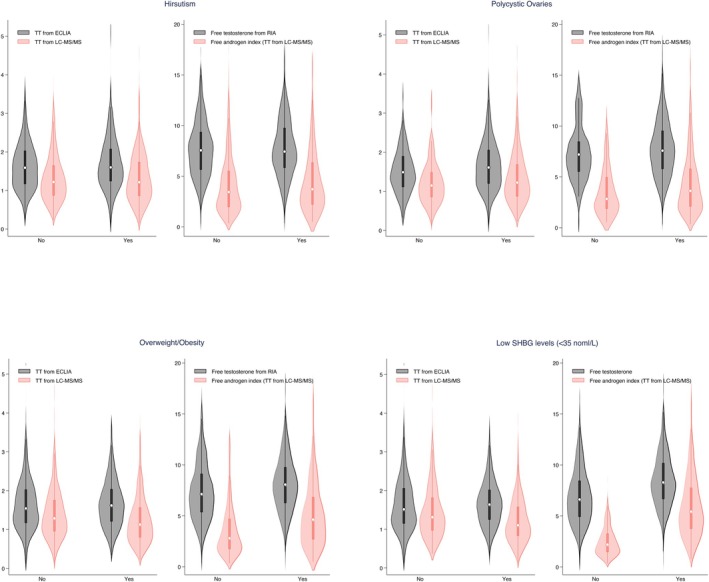
Assessment of TT, FT, and FAI across different clinical phenotypes. The violin plot showed the distribution of assay‐specific total testosterone (TT), free testosterone (FT), and free androgen index (FAI) across various clinical phenotypes, including hirsutism, polycystic ovaries, overweight/obesity, and low sex hormone‐binding globulin (SHBG) levels. The central thick bar denotes the interquartile range, while the white dot signifies the median value. Hirsutism was defined as modified Ferriman‐Gallwey score ≥ 5. Polycystic ovaries were diagnosed by transvaginal ultrasound when at least one ovary had a volume of > 10 mL or there were 12 or more follicles measuring 2–9 mm in diameter. Overweight/obesity was defined as BMI of ≥ 24 kg/m^2^. Low SHBG levels were set as below 35 nmol/L.

The Passing‐Bablok regression analysis resulted in the following linear equation: y (TT from ECLIA) = 0.26 + 1.04*TT (from LC–MS/MS), with the 95% CI for the intercept ranging from 0.18 to −0.33 and for slope from 0.98 to 1.10 (Figure 1A). This indicated a small constant difference between the two methods. The Bland–Altman plots demonstrated that the mean percentage difference (bias) was 23.8% (95% limits of agreement −44.2% to 91.9%) between the ECLIA and LC–MS/MS (Figure 1B). A Pearson correlation coefficient of 0.69 (*p* < 0.001) was observed between ECLIA and LC–MS/MS measurements. To further investigate whether the discrepancy in TT levels between the two assessments varied across different clinical phenotypes, including hirsutism, polycystic ovary morphology (PCOM), overweight/obesity and low SHBG level (< 35 nmol/L), the median TT levels from both methods, as well as free testosterone and FAI, were plotted. Overall, TT levels measured by ECLIA were consistently higher than those measured by LC–MS/MS regardless of the clinical phenotypes considered (Figure 2).

**FIGURE 3:**
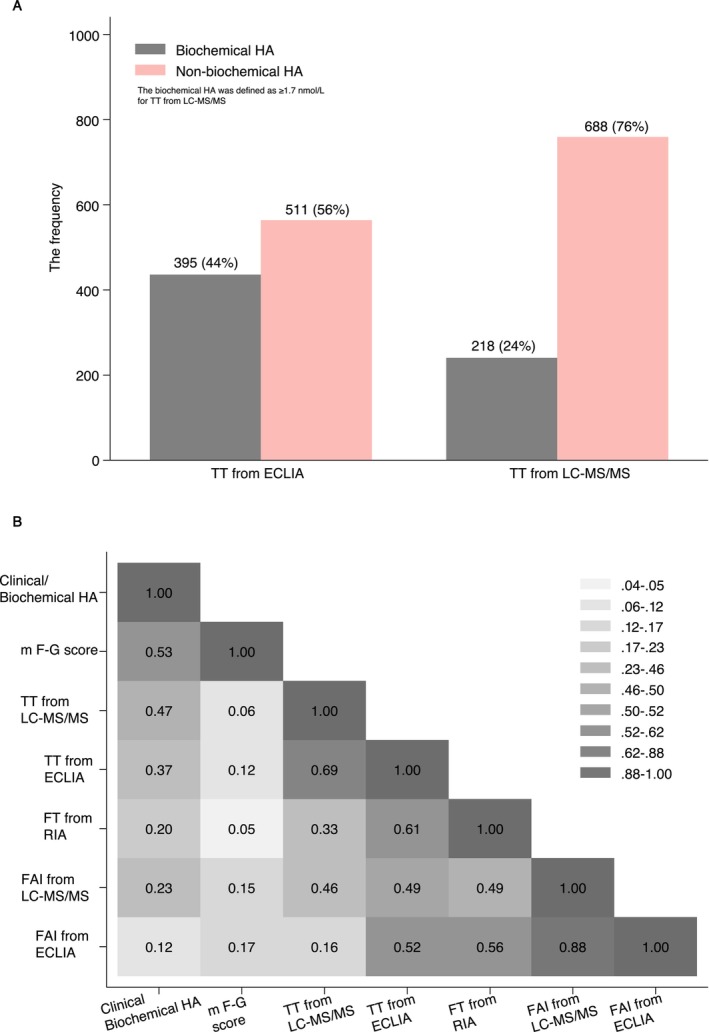
Correlation and comparison between assay‐specific TT measurement and other androgen index. (A) Comparison of biochemical hyperandrogenism determined by assay‐specific TT levels. (B) Correlation matrix of assay‐specific TT measurement and associated FAI, modified Ferriman‐Gallwey (mF‐G) score, and clinical/biochemical hyperandrogenism.

When biochemical hyperandrogenism (HA) was defined as TT levels of ≥ 1.7 nmol/L as measured by LC–MS/MS method, a significantly higher proportion of patients were identified as having biochemical HA when using ECLIA (44.0% vs. 24.0%, *p* < 0.001) in comparison to LC–MS/MS (Figure 3A). However, the correlations of TT levels measured by the two methods with biochemical HA were weak; a similar pattern was also observed for FT and FAI (Figure 3B).

Although TT levels measured by both methods were associated with higher amenorrhea (RR 1.69, 95% CIs 1.25–2.29; 1.52, 1.31–1.78, respectively) and NICU admission (RR 2.05, 95% CIs 1.03–4.07; RR 2.22, 95% CIs 1.07–4.63) and lower rates of ovulation (RR 0.61, 95% CIs 0.47–0.80; RR 0.59, 95% CIs 0.45–0.77), TT measured only by LC–MS/MS was associated with overweight/obesity (RR 0.69, 95% CIs 0.56–0.86), metabolic syndrome (RR 0.69, 95% CIs 0.60–099), and preterm labor (RR 2.25, 95% CIs 1.17–5.55) (Table S1).

Given a threshold of 1.7 nmol/L for TT as measured by LC–MS/MS, patients were stratified into three groups: those with TT < 1.7 nmol/L (*n* = 511, 56.4%) as determined by both methods, those with TT ≥ 1.7 nmol/L by ECLIA but < 1.7 nmol/L by LC–MS/MS (*n* = 202, 22.3%), and those with ≥ 1.7 nmol/L by both methods (*n* = 193, 21.3%). In comparison with patients with TT < 1.7 nmol/L, no significant differences were observed in clinical characteristics or fertility and neonatal outcomes among patients identified with TT levels of ≥ 1.7 nmol/L determined by ECLIA and < 1.7 nmol/L by LC–MS/MS. Conversely, patients identified with TT levels of ≥ 1.7 nmol/L by LC–MS/MS demonstrated significantly higher risks of amenorrhea (RR 2.11, 95% CIs 1.29–3.44), preterm labor (RR 4.07, 95% CIs 1.05–15.77), and NICU (RR 3.20, 95% CIs 1.02–10.03), and lower rates of ovulation (RR 0.52, 95% CIs 0.34–0.79) (Table 2).

**TABLE 2:** The clinical presentation and fertility outcomes in subgroups.

Outcomes	TT levels from ECLIA and LC–MS/MS (nmol/L)		
	Both < 1.7 (*n* = 511)	LC–MS/MS < 1.7 but ECLIA ≥ 1.7 (*n* = 202)	Both ≥ 1.7 (*n* = 193)
Clinical characteristic			
Amenorrhea	Reference	1.17 (0.69, 1.99)	2.11 (1.29, 3.44)
Hirsutism	Reference	0.93 (0.63, 1.36)	1.24 (0.86, 1.80)
Acne	Reference	1.28 (0.90, 1.82)	1.19 (0.83, 1.69)
Overweight/obesity	Reference	0.59 (0.22, 1.65)	0.74 (0.53, 1.05)
HOMA‐IR index (> 2.69)	Reference	1.74 (1.25, 2.42)	0.81 (0.58, 1.14)
Acanthosis nigricans (> 1)	Reference	0.76 (0.49, 1.18)	0.97 (0.62, 1.52)
Metabolic syndrome	Reference	1.29 (0.84, 1.20)	1.03 (0.63, 1.67)
Fertility outcomes^a^			
Ovulation	Reference	0.81 (0.53, 1.24)	0.52 (0.34, 0.79)
Conception	Reference	0.89 (0.61, 1.27)	0.89 (0.62, 1.27)
Clinical pregnancy	Reference	0.82 (0.54, 1.23)	0.88 (0.59, 1.31)
Live birth	Reference	0.78 (0.51, 1.19)	0.89 (0.59, 1.34)
Pregnancy loss	Reference	1.34 (0.71, 2.50)	1.12 (0.60, 2.09)
Obstetric outcomes^a^			
Threatened miscarriage	Reference	1.12 (0.57, 2.22)	1.07 (0.53, 2.14)
Gestational diabetes mellitus	Reference	1.04 (0.31, 3.51)	0.85 (0.22, 3.22)
Preterm labor	Reference	2.75 (0.69, 10.96)	4.07 (1.05, 15.77)
NICU	Reference	1.89 (0.52, 6.94)	3.20 (1.02, 10.03)

*Note:* Relative risk (RR) and 95% Confidence interval are presented. TT level ≥ 1.7 nmol/L was determined by LC–MS/MS.

^a^ Adjustment by age, BMI, and treatment.

## 4 Discussion

In this study, we found that TT levels quantified via ECLIA were markedly elevated in comparison to those determined by LC–MS/MS. When compared to the LC–MS/MS method, the proportion of patients diagnosed with biochemical HA, as identified by TT levels measured by ECLIA, exhibited a 20% augmentation. Furthermore, patients with TT levels of ≥ 1.7 nmol/L, identified exclusively through LC–MS/MS, demonstrated an increased risk of amenorrhea, preterm labor, anovulation, and NICU admission, while these increased risks were not observed among patients with TT ≥ 1.7 nmol/L quantified solely by ECLIA.

Given that LC–MS/MS has been advocated for measuring TT to identify the HA in the diagnosis and management of PCOS [13], particularly for establishing a diagnosis of PCOS, our study aligns with previous studies [12, 22]. Serum TT levels measured by ECLIA were higher than those measured by LC–MS, with a mean percentage difference of 23.8%. This discrepancy between the methodologies proves to be consistently reliable across a spectrum of clinical phenotypes and in scenarios of low TT levels. In this study, serum TT measured by ECLIA was, on average, higher than that measured by LC–MS/MS, with wide limits of agreement between paired measurements from the same samples. This finding is consistent with a prior report indicating that the Roche ECLIA routine testosterone immunoassay may overestimate TT in women and children when compared with a LC–MS/MS method, particularly at low TT concentrations [23]. At these levels, even modest, non‐specific binding or cross‐reactivity can materially contribute to the reported concentration. A key contributor to the observed widespread dispersion is likely analytical specificity. Immunoassays rely on antibody recognition and are therefore vulnerable to cross‐reactivity with structurally related endogenous steroids and their metabolites, as well as to other sample‐specific interferences (e.g., heterophile antibodies or other matrix constituents). Such interferences are not necessarily constant across individuals, which can plausibly explain both a net positive bias and the marked dispersion observed in Bland–Altman analyses. In contrast, LC–MS/MS improves analytical specificity by combining chromatographic separation with mass‐selective detection, reducing susceptibility to cross‐reactivity that can affect immunoassays in low TT samples. Differences in calibration/standardization may contribute to method disagreement; however, both platforms used commercial calibrators traceable to higher‐order reference materials. A predominantly calibration‐driven bias would be expected to present as a relatively uniform offset across the measurement range. The broad relative limits of agreement observed here suggest that sample‐dependent effects, rather than calibration alone, likely account for a substantial portion of the variability between the two methods. LC–MS/MS is commonly regarded as the reference approach for female TT measurement because it can achieve greater selectivity and more reliable quantification at low concentrations when appropriately validated. In the present work, matrix effects inherent to electrospray ionization were addressed using a stable isotope–labeled internal standard (^13^C_3_ testosterone). Our method was validated in accordance with CLSI guideline 62A for LC–MS methods, and our testing laboratory, accredited by NATA, has maintained satisfactory RCPA external quality assurance performance. Recent studies confirm that well‐validated LC–MS/MS assays provide comparable results across platforms for steroids, including TT, even at low concentrations [24]. Collectively, these factors support the interpretation that the higher ECLIA TT values and the wide method disagreement primarily reflect limitations of immunoassay specificity and low‐concentration performance in female samples, rather than actual biological differences.

At a diagnostic threshold of 1.7 nmol/L, this variance leads to a 20% augmentation in the identification of biochemical HA cases. Coupled with advancements in ultrasonographic technologes [25, 26], this has, to a significant degree, fueled the escalating prevalence of PCOS diagnosis over recent decades. Such findings reinforce the argument that biochemical HA should preferably be identified through androgen markers quantified by LC–MS/MS. In light of the fact that a PCOS diagnosis can exert adverse impacts on the psychiatric well‐being of affected individuals, manifesting as heightened incidences of moderate to severe depressive and anxiety symptoms [27] and body‐image distress [28], a cautious approach is warranted in diagnosing PCOS, particularly when HA serves as a mandatory diagnostic criterion. Because of the requirement for advanced equipment, skilled operators, and the costly nature of LC–MS/MS, the ECLIA method remains prevalent in routine clinical settings due to its rapid and cost‐effective measurements. In setting where mass spectrometry is not accessible, FAI can be employed as an alternative. This implies that the simultaneous quantification of TT and SHBG to calculate the FAI serves as a viable substitute for LC–MS/MS measurement. Considering the significant correlation between TT and SHBG [29], it is imperative to have commercial Kits with high sensitivity and specificity for SHBG. In addition, conditions that influence the SHBG levels, such as obesity [30], should be taken into account.

Previously, androgen markers predictive of pregnancy outcomes have been reported in infertile patients with PCOS undergoing ovulation, including TT, FT, and FAI [31]. Notably, TT levels, as determined only by LC–MS/MS, were significantly associated with preterm labor and NICU admission. Furthermore, we found that TT levels ≥ 1.7 nmol/L, extensively quantified by LC–MS/MS, were significantly correlated with the risks of anovulation, preterm labor, and NICU admission. These findings offer more prognostic information on clinical outcomes compared to ECLIA. According to the recently issued 2023 International Evidence‐Based Guideline for the assessment and management of PCOS, the use of mass spectrometry to evaluate biochemical HA through TT or FT is also recommended. Considering that LC–MS/MS technique recently can be assessable in partial territory hospitals in mainland China, due to expensive initial equipment and maintenance cost and need for specialized technicians, thereby it is difficult to generalize as a routine application, particularly in low‐income countries where access to mass spectrometry is limited. Several challenges should be addressed before this method can be widely implemented for clinical diagnostics, including the lack of assay standardization, the unavailability of commercial quality control material, and the high technical proficiency required of laboratory technicians [32]. Given the increasing global incidence and burden of PCOS [33], there is an eager need for studies aimed at simplifying and standardizing the LC–MS/MS procedure and reducing costs, thereby providing better support in the management of PCOS. Additionally, a highly accurate LC–MS/MS method with a limit of detection of 9.71 pmol/L (0.28 ng/dL), as previously reported [34], may lead to the detection of hypoandrogenism in this population due to the absence of normative data on TT levels across the lifespan. This could unnecessarily alarm both physicians and patients, potentially resulting in testosterone therapy, which carries potential risks and hazards, such as cardiovascular diseases [35].

The primary strength of this study lies in the simultaneous measurement of TT by both LC–MS/MS and ECLIA in a large cohort of infertile patients diagnosed with PCOS. Furthermore, this study thoroughly evaluated the associations of TT with clinical phenotypes as well as fertility and obstetric outcomes. Consequently, we distinguished a subgroup at genuine risk of adverse fertility outcomes in infertile women with PCOS, indicating patients with TT > 1.7 nmol/L measured by LC–MS/MS at risk of adverse fertility outcomes in infertile women with PCOS. However, this study also had several limitations. Firstly, the samples utilized to assess androgen profiles were derived from the PCOSAct study. The assessments of TT were confined to baseline measurement, rather than post‐treatment levels or changes relative to baseline. The latter approach would likely yield more comprehensive and detailed insights. Additionally, the relationships between TT and some infrequent clinical outcomes, such as preterm labor, may lack the requisite statistical power for generalizability across all PCOS populations. This is attributed to the relatively small number of events, as well as variations in ethnicity and diagnostic criteria. Finally, there was no formal cost‐effectives analysis in this study. Based on cost of TT measured by LC–MS/MS was roughly estimated over 10‐fold higher than by ECLIA. A rigorous and formal cost‐effectiveness analysis on approximate per‐test cost of LC–MS/MS vs. ECLIA, initial equipment and maintenance cost, need for specialized personal, turnaround time and medical cost for overdiagnosis and treatment, is needed to be conducted across different countries on healthy care systems.

## 5 Conclusions

In summary, our findings indicated that LC–MS/MS refined the diagnosis of biochemical hyperandrogenism and better identified the subgroup at genuine risk of adverse fertility outcomes in infertile women with PCOS.

## Funding

This study was funded by the National key R&D Program of China (2019YFC1709500), the Health and Medical Research Fund, Food and Health Bureau (06171026), the Science & Technology Department of Guizhou province (ZK[2022]‐428), PhD funding in the Affiliated Hospital Guizhou Medical University (gyfybsky‐2021‐24). The study's funder had no role in the study design, collection, analysis, and interpretation of the data; in the writing of the manuscript; and in the decision to submit the article for publication. The authors had full access to all the data in the study and had final responsibility for the decision to submit for publication.

## Ethics Statement

The study was approved by Ethics Committee at the First Affiliated Hospital of Heilongjiang. University of Chinese Medicine (IRB 2010HZYLL‐010) and registered on chictr.org.cn (ChiCTR‐TRC‐12002081).

## Conflicts of Interest

All authors declare no conflicts of interest. B.W.J.M. is supported by a NHMRC Investigator grant (GNT1176437); consultancy for ObsEva and Merck; and travel support from Merck. The remaining authors report no conflict of interest.

## Supporting information


**Table S1:** Association of baseline TT measured by ECLIA and LC–MS/MS with clinical presentations and outcomes.

## Data Availability

The data that support the findings of this study are available from the corresponding author upon reasonable request.

## References

[1] H. J. Teede , C. T. Tay , J. Laven , et al., “Recommendations From the 2023 International Evidence‐Based Guideline for the Assessment and Management of Polycystic Ovary Syndrome,” Fertility and Sterility 120, no. 4 (2023): 767–793, 10.1016/j.fertnstert.2023.07.025. 37589624

[2] H. F. Escobar‐Morreale , “Polycystic Ovary Syndrome: Definition, Aetiology, Diagnosis and Treatment,” Nature Reviews. Endocrinology 14, no. 5 (2018): 270–284, 10.1038/nrendo.2018.24. 29569621

[3] R. Li , Q. Zhang , D. Yang , et al., “Prevalence of Polycystic Ovary Syndrome in Women in China: A Large Community‐Based Study,” Human Reproduction 28, no. 9 (2013): 2562–2569, 10.1093/humrep/det262. 23814096

[4] R. L. Rosenfield and D. A. Ehrmann , “The Pathogenesis of Polycystic Ovary Syndrome (PCOS): The Hypothesis of PCOS as Functional Ovarian Hyperandrogenism Revisited,” Endocrine Reviews 37, no. 5 (2016): 467–520, 10.1210/er.2015-1104. 27459230 PMC5045492

[5] S. Palomba , T. T. Piltonen , and L. C. Giudice , “Endometrial Function in Women With Polycystic Ovary Syndrome: A Comprehensive Review,” Human Reproduction Update 27, no. 3 (2021): 584–618, 10.1093/humupd/dmaa051. 33302299

[6] S. Risal , Y. Pei , H. Lu , et al., “Prenatal Androgen Exposure and Transgenerational Susceptibility to Polycystic Ovary Syndrome,” Nature Medicine 25, no. 12 (2019): 1894–1904, 10.1038/s41591-019-0666-1. 31792459

[7] A. Sharma and C. K. Welt , “Practical Approach to Hyperandrogenism in Women,” Medical Clinics of North America 105, no. 6 (2021): 1099–1116, 10.1016/j.mcna.2021.06.008. 34688417 PMC8548673

[8] J. L. Shea , P. Y. Wong , and Y. Chen , “Free Testosterone: Clinical Utility and Important Analytical Aspects of Measurement,” in Adv Clin Chem, vol. 63 (Elsevier, 2014), 59–84, 10.1016/b978-0-12-800094-6.00002-9. 24783351

[9] H. I. Jansen , A. E. van Herwaarden , H. J. Huijgen , et al., “Lower Accuracy of Testosterone, Cortisol, and Free T4 Measurements Using Automated Immunoassays in People Undergoing Hemodialysis,” Clinical Chemistry and Laboratory Medicine 61, no. 8 (2023): 1436–1445, 10.1515/cclm-2022-1133. 36877870

[10] A. D. Bizuneh , A. E. Joham , H. Teede , et al., “Evaluating the Diagnostic Accuracy of Androgen Measurement in Polycystic Ovary Syndrome: A Systematic Review and Diagnostic Meta‐Analysis to Inform Evidence‐Based Guidelines,” Human Reproduction Update 31, no. 1 (2024): 48–63, 10.1093/humupd/dmae028. PMC11696697 39305127

[11] Y. Yang , N. Ouyang , Y. Ye , et al., “The Predictive Value of Total Testosterone Alone for Clinical Hyperandrogenism in Polycystic Ovary Syndrome,” Reproductive Biomedicine Online 41, no. 4 (2020): 734–742, 10.1016/j.rbmo.2020.07.013. 32912651

[12] D. J. Handelsman , H. J. Teede , R. Desai , R. J. Norman , and L. J. Moran , “Performance of Mass Spectrometry Steroid Profiling for Diagnosis of Polycystic Ovary Syndrome,” Human Reproduction 32, no. 2 (2017): 418–422, 10.1093/humrep/dew328. 27999117

[13] H. J. Teede , M. L. Misso , M. F. Costello , et al., “Recommendations From the International Evidence‐Based Guideline for the Assessment and Management of Polycystic Ovary Syndrome,” Human Reproduction 33, no. 9 (2018): 1602–1618, 10.1093/humrep/dey256. 30052961 PMC6112576

[14] X. K. Wu , E. Stener‐Victorin , H. Y. Kuang , et al., “Effect of Acupuncture and Clomiphene in Chinese Women With Polycystic Ovary Syndrome: A Randomized Clinical Trial,” JAMA 317, no. 24 (2017): 2502–2514, 10.1001/jama.2017.7217. 28655015 PMC5815063

[15] B. F. Zhou , “Effect of Body Mass Index on All‐Cause Mortality and Incidence of Cardiovascular Diseases–Report for Meta‐Analysis of Prospective Studies Open Optimal Cut‐Off Points of Body Mass Index in Chinese Adults,” Biomedical and Environmental Sciences 15, no. 3 (2002): 245–252. 12500665

[16] X. Zhao , R. Ni , L. Li , et al., “Defining Hirsutism in Chinese Women: A Cross‐Sectional Study,” Fertility and Sterility 96, no. 3 (2011): 792–796, 10.1016/j.fertnstert.2011.06.040. 21762890

[17] J. K. L. Tan , J. Tang , K. Fung , et al., “Development and Validation of a Comprehensive Acne Severity Scale,” Journal of Cutaneous Medicine and Surgery 11, no. 6 (2007): 211–216, 10.2310/7750.2007.00037. 18042334

[18] J. P. Burke , D. E. Hale , H. P. Hazuda , and M. P. Stern , “A Quantitative Scale of Acanthosis Nigricans,” Diabetes Care 22, no. 10 (1999): 1655–1659, 10.2337/diacare.22.10.1655. 10526730

[19] C. D. Society , “Guidelines for the Prevention and Treatment of Type 2 Diabetes in China (2017 Edition),” Chinese Journal of Diabetes Mellitus 10 (2018): 4–67.

[20] H. Cs and L. Eyk , “Female Androgens Profile by MS for PCOS Patients,” Clinica Chimica Acta 2010 (2010): 901.

[21] G. Zou , “A Modified Poisson Regression Approach to Prospective Studies With Binary Data,” American Journal of Epidemiology 159, no. 7 (2004): 702–706, 10.1093/aje/kwh090. 15033648

[22] G. Grassi , E. Polledri , S. Fustinoni , et al., “Hyperandrogenism by Liquid Chromatography Tandem Mass Spectrometry in PCOS: Focus on Testosterone and Androstenedione,” Journal of Clinical Medicine 10, no. 1 (2020): E119, 10.3390/jcm10010119. PMC7795755 33396396

[23] V. Moal , E. Mathieu , P. Reynier , Y. Malthièry , and Y. Gallois , “Low Serum Testosterone Assayed by Liquid Chromatography‐Tandem Mass Spectrometry. Comparison With Five Immunoassay Techniques,” Clinica Chimica Acta 386, no. 1–2 (2007): 12–19, 10.1016/j.cca.2007.07.013. 17706625

[24] V. Braun , U. Ceglarek , A. Gaudl , et al., “Evaluation of Five Multisteroid LC–MS/MS Methods Used for Routine Clinical Analysis: Comparable Performance Was Obtained for Nine Analytes,” Clinical Chemistry and Laboratory Medicine 62, no. 5 (2024): 900–910, 10.1515/cclm-2023-0847. 38038605

[25] M. A. Skiba , R. M. Islam , R. J. Bell , and S. R. Davis , “Understanding Variation in Prevalence Estimates of Polycystic Ovary Syndrome: A Systematic Review and Meta‐Analysis,” Human Reproduction Update 24, no. 6 (2018): 694–709, 10.1093/humupd/dmy022. 30059968

[26] D. Dewailly , H. Gronier , E. Poncelet , et al., “Diagnosis of Polycystic Ovary Syndrome (PCOS): Revisiting the Threshold Values of Follicle Count on Ultrasound and of the Serum AMH Level for the Definition of Polycystic Ovaries,” Human Reproduction 26, no. 11 (2011): 3123–3129, 10.1093/humrep/der297. 21926054

[27] L. G. Cooney , I. Lee , M. D. Sammel , and A. Dokras , “High Prevalence of Moderate and Severe Depressive and Anxiety Symptoms in Polycystic Ovary Syndrome: A Systematic Review and Meta‐Analysis,” Human Reproduction 32, no. 5 (2017): 1075–1091, 10.1093/humrep/dex044. 28333286

[28] S. Alur‐Gupta , A. Chemerinski , C. Liu , et al., “Body‐Image Distress Is Increased in Women With Polycystic Ovary Syndrome and Mediates Depression and Anxiety,” Fertility and Sterility 112, no. 5 (2019): 930–938, 10.1016/j.fertnstert.2019.06.018. 31395311 PMC6858949

[29] J. F. Dunn , B. C. Nisula , and D. Rodbard , “Transport of Steroid Hormones: Binding of 21 Endogenous Steroids to Both Testosterone‐Binding Globulin and Corticosteroid‐Binding Globulin in Human Plasma,” Journal of Clinical Endocrinology and Metabolism 53, no. 1 (1981): 58–68, 10.1210/jcem-53-1-58. 7195404

[30] X. Qu and R. Donnelly , “Sex Hormone‐Binding Globulin (SHBG) as an Early Biomarker and Therapeutic Target in Polycystic Ovary Syndrome,” International Journal of Molecular Sciences 21, no. 21 (2020): E8191, 10.3390/ijms21218191. PMC7663738 33139661

[31] H. Kuang , S. Jin , K. R. Hansen , et al., “Identification and Replication of Prediction Models for Ovulation, Pregnancy and Live Birth in Infertile Women With Polycystic Ovary Syndrome,” Human Reproduction 30, no. 9 (2015): 2222–2233, 10.1093/humrep/dev182. 26202922 PMC4542721

[32] S. N. Thomas , D. French , P. J. Jannetto , B. A. Rappold , and W. A. Clarke , “Liquid Chromatography–Tandem Mass Spectrometry for Clinical Diagnostics,” Nature Reviews Methods Primers 2, no. 1 (2022): 96, 10.1038/s43586-022-00175-x. 36532107 PMC9735147

[33] S. Safiri , M. Noori , S. A. Nejadghaderi , et al., “Prevalence, Incidence and Years Lived With Disability due to Polycystic Ovary Syndrome in 204 Countries and Territories, 1990‐2019,” Human Reproduction 37, no. 8 (2022): 1919–1931, 10.1093/humrep/deac091. 35586937

[34] Y. Wang , G. D. Gay , J. C. Botelho , S. P. Caudill , and H. W. Vesper , “Total Testosterone Quantitative Measurement in Serum by LC‐MS/MS,” Clinica Chimica Acta 436 (2014): 263–267, 10.1016/j.cca.2014.06.009. PMC4743493 24960363

[35] A. Traish , A. T. Guay , R. F. Spark , and Testosterone Therapy in Women Study Group , “Are the Endocrine Society's Clinical Practice Guidelines on Androgen Therapy in Women Misguided? A Commentary,” Journal of Sexual Medicine 4, no. 5 (2007): 1223–1234, 10.1111/j.1743-6109.2007.00584.x. 17727347

