# Supplementary material for: Total Testosterone Measured by Liquid Chromatograph‐Tandem Mass Spectrometry Refines Diagnosis of Biochemical Hyperandrogenism and Better Identifies Subgroup at Genuine Risk of Adverse Fertility Outcomes in Women With Polycystic Ovary Syndrome
Source: Reprod Med Biol. 2026 Jan 28;25(1):e70013. doi: 10.1002/rmb2.70013 (PMC12848768; doi:10.1002/rmb2.70013)
Supplement: Supplementary file 1 — Table S1: Association of baseline TT measured by ECLIA and LC–MS/MS with clinical presentations and outcomes. [file RMB2-25-e70013-s001.docx]

| **Table S1.** Association of baseline TT measured by ECLIA and LC-MS/MS with clinical presentations and outcomes | | | | | | |
| --- | --- | --- | --- | --- | --- | --- |
| Clinical presentations and outcomes | TT | |  | | TT to SHBG ratio | |
|  | ECLIA | LC-MS/MS | | ECLIA | | LC-MS/MS |
| **Clinical presentations** |  |  | |  | |  |
| Clinical/biochemical HA | 3.97 (3.10, 5.14) | 9.30 (6.67, 12.97) | | 1.17 (1.11, 1.23) | | 1.17 (1.11, 1.23) |
| Amenorrhea | 1.69 (1.25, 2.29) | 1.52 (1.31, 1.78) | | 1.02 (0.97, 1.10) | | 1.06 (1.02, 1.09) |
| Hirsutism | 1.14 (0.90, 1.43) | 1.04 (0.83, 1.32) | | 1.04 (1.01, 1.08) | | 1.05 (1.00, 1.11) |
| Acne | 1.27 (1.02, 1.58) | 1.05 (0.84, 1.32) | | 1.02 (0.99, 1.05) | | 1.01 (0.96, 1.06) |
| Overweight/obesity | 1.15 (0.94, 1.42) | 0.69 (0.56, 0.86) | | 1.22 (1.17, 1.27) | | 1.11 (1.06, 1.16) |
| Insulin resistance | 1.01 (0.90, 1.37) | 0.87 (0.73, 1.02) | | 1.18 (1.14, 1.22) | | 1.18 (1.12, 1.24) |
| Acanthosis score >1 | 1.07 (0.82, 1.38) | 0.76 (0.57, 1.01) | | 1.03 (0.99, 1.13) | | 1.02 (0.99, 1.08) |
| PCO morphology | 1.01 (0.79, 1.30) | 1.29 (0.91, 1.83) | | 1.11 (1.02, 1.20) | | 1.11 (1.02, 1.20) |
| Metabolic syndrome | 1.01 (0.79, 1.30) | 0.69 (0.60, 0.99) | | 1.16 (1.11, 1.20) | | 1.05 (1.01, 1.10) |
| **Fertility outcomes*** |  |  | |  | |  |
| Ovulation | 0.61 (0.47, 0.80) | 0.59 (0.45, 0.77) | | 0.92 (0.89, 0.96) | | 0.86 (0.82, 0.91) |
| Conception | 0.83 (066, 1.05) | 0.91 (0.72, 1.58) | | 0.95 (0.91, 0.99) | | 0.93 (0.88, 0.98) |
| Clinical pregnancy | 0.78 (0.60, 1.01) | 0.91 (0.70, 1.18) | | 0.92 (0.87, 0.96) | | 0.89 (0.83, 0.95) |
| Live birth | 0.79 (0.61, 1.03) | 0.94 (0.72, 1.23) | | 0.92 (0.87, 0.96) | | 0.89 (0.84, 0.96) |
| Pregnancy loss | 1.22 (0.82, 1.81) | 0.98 (0.65, 1.47) | | 1.09 (1.02, 1.17) | | 1.10 (0.99, 1.20) |
| **Obstetric outcomes*** |  |  | |  | |  |
| Threatened miscarriage | 0.83 (0.53, 1.29) | 0.83 (0.53, 1.30) | | 1.01 (0.94, 1.08) | | 1.00 (0.90, 1.10) |
| Gestational diabetes | 0.93 (0.41, 2.11) | 0.84 (0.36, 1.99) | | 1.07 (0.96, 1.19) | | 1.14 (0.98, 1.33) |
| Preterm labor | 1.84 (0.89, 3.81) | 2.55 (1.17, 5.55) | | 1.12 (0.97, 1.29) | | 1.26 (1.03, 1.53) |
| NICU admission | 2.05 (1.03, 4.07) | 2.22 (1.07, 4.63) | | 1.11 (0.97, 1.27) | | 1.22 (1.00, 1.48) |
| Abbreviation: ECLIA: Electro-chemiluminescent immunoassays; LC-MS/MS: liquid chromatograph-tandem mass spectrometry; HA: hyperandrogenism; NICU: neonatal intensive care unit; TT: total testosterone; SHBG: sex hormone blinding globulin; PCO: polycystic ovary.  Relative risk (RR) and 95% Confidence intervals (CIs) are presented. RR refers to patients with positive status of clinical phenotypes and fertility outcomes relative to its counterparts.  *Adjustment by age, BMI, and treatment. | | | | | | |
